# Association between the triglyceride glucose-body mass index and the risk of incident type 2 diabetes: a systematic review and meta-analysis

**DOI:** 10.3389/fnut.2026.1908337

**Published:** 2026-09-17

**Authors:** Bo Dai, Hang Yu, Haotian Qi, Yang Xiao, Yanxu Chen, Zhenru Liu, Jieyu Zhang, Jiali Pei, Yue Zhang, Rui Chen, Zheng Nan, Jian Sun

**Affiliations:** 1 Changchun University of Chinese Medicine, Changchun, China; 2 College of Science and Technology Changchun, Changchun, China; 3 Jilin Cancer Hospital, Changchun, China; 4 The Affiliated Hospital of Changchun University of Chinese Medicine, Changchun, China

**Keywords:** IR, meta-analysis, systematic review, triglyceride glucose-body mass index, TyG-BMI, type 2 diabetes

## Abstract

**Objective:**

The rising prevalence of type 2 diabetes (T2D) has created a huge global public health burden. Although triglyceride glucose-body mass index (TyG-BMI) has been associated with incident T2D risk in observational studies, the overall evidence remains to be systematically evaluated.

**Methods:**

This study systematically searched Chinese databases (China National Knowledge Infrastructure, Wanfang Data, and China Science and Technology Journal Database) and English databases (PubMed, Web of Science, Cochrane Library, Embase), with the search period covering from the establishment of each database to May 1, 2026. Literature screening, data extraction, and quality assessment were independently performed by two reviewers using the Newcastle-Ottawa Scale (NOS) for study quality evaluation. Meta-analysis was conducted using Stata 16.0 and Review Manager 5.4.1 software: Effect sizes were pooled using a random-effects model, with results reported as hazard ratios (HR) and 95% confidence intervals (CI). Subgroup analyses by the proportion of male participants, follow-up duration, and sample size explored sources of heterogeneity. Sensitivity analyses using exclusion of individual studies validated result robustness. Finally, risk of publication bias was assessed via funnel plot symmetry testing and Egger’s regression.

**Results:**

A total of six cohort studies involving 176,127 participants were included in this meta-analysis. For continuous-variable analysis, four studies reporting hazard ratios (HRs) per 1-standard deviation (SD) increase in TyG-BMI were included. The pooled analysis demonstrated that each 1-SD increase in TyG-BMI was significantly associated with an increased risk of incident type 2 diabetes (HR = 1.68, 95% CI: 1.50–1.88, I^2^ = 87%). Subgroup analyses according to sex distribution, follow-up duration, and sample size revealed no significant differences in effect estimates, except that sample size may partially contribute to between-study heterogeneity. For categorical-variable analysis, six studies showed that higher TyG-BMI categories were associated with an increased risk of incident type 2 diabetes (HR = 3.35, 95% CI: 2.24–5.03, I^2^ = 89%). Subgroup analyses indicated that follow-up duration significantly modified the association, with stronger effects observed in studies with follow-up duration < 7 years (HR = 6.22, 95% CI: 3.49–11.10) compared with those with follow-up duration ≥ 7 years (HR = 2.50, 95% CI: 1.77–3.53). Sensitivity analyses demonstrated that the pooled estimates remained stable after sequential exclusion of individual studies. Funnel plots and Egger’s tests showed no statistically significant evidence of publication bias.

**Conclusion:**

This meta-analysis reveals a possible association between elevated TyG-BMI and higher risk of incident T2D in the general population. With the advantages of convenience and low cost, TyG-BMI may represent a potential marker associated with T2D risk stratification, although further studies are required to determine its clinical applicability. Due to the limited number of eligible studies, the assessment of publication bias was insufficient. Therefore, our findings should be regarded as preliminary and require validation in future large-scale investigations.

**Systematic review registration:**

https://www.crd.york.ac.uk/PROSPERO/view/CRD420251232373, identifier CRD420251232373.

## 1 Introduction

Type 2 diabetes (T2D) represents a major global public health challenge. The International Diabetes Federation (IDF) predicts that the global number of people with diabetes will reach 783 million by 2045, with T2D accounting for more than 90% of all diabetes cases (1). Beyond its high prevalence, T2D imposes substantial health and socioeconomic burdens worldwide. It is a major contributor to cardiovascular disease, renal failure, blindness, and lower-limb amputation, resulting in millions of premature deaths and considerable healthcare expenditure each year (2, 3). Therefore, early identification of individuals at increased risk of T2D is important for implementing timely preventive strategies. However, currently available risk assessment approaches, including demographic characteristics, anthropometric indicators, metabolic markers, and composite risk scores, have certain limitations. Fasting blood glucose is widely used in clinical practice but mainly reflects current glycemic status and may not fully capture early metabolic abnormalities (4). Body mass index (BMI) reflects overall obesity status but does not directly reflect metabolic dysfunction associated with insulin resistance. In addition, some composite risk scores require multiple parameters or rely on self-reported information, which may limit their applicability in large-scale population assessment (5, 6).

The triglyceride-glucose (TyG) index is calculated as ln [triglycerides (mg/dL) × glucose (mg/dL)/2]. It has been widely investigated as a simple surrogate marker of insulin resistance (IR), a key pathological mechanism underlying T2D development (7, 8). However, the TyG index alone cannot fully explain obesity, a major modifiable risk factor for IR and T2D. A clear dose-response relationship exists between obesity and disease risk (9). To address this limitation, the triglyceride-glucose-body mass index (TyG-BMI) was proposed by integrating the metabolic information provided by TyG with the anthropometric characteristics reflected by BMI. This combined index may provide a more comprehensive assessment of metabolic abnormalities associated with T2D risk (10). Although previous observational studies have reported an association between higher TyG-BMI and increased risk of incident T2D, variations remain across populations, follow-up durations, and analytical approaches (11, 12).

To date, no meta-analysis has systematically evaluated the association between TyG-BMI and incident T2D. Therefore, this systematic review and meta-analysis aimed to synthesize available cohort evidence, quantify the association between TyG-BMI and incident T2D, and explore potential sources of heterogeneity, thereby providing evidence regarding the potential role of TyG-BMI in future T2D risk assessment.

## 2 Methods

This meta-analysis and systematic review followed the PRISMA guidelines and was registered in the International Prospective Registration of Systematic Reviews (PROSPERO) with registration number CRD420251232373. The PRISMA 2020 checklist is available in Supplementary Material 1.

### 2.1 Database and search strategy

A comprehensive literature search was conducted in both English and Chinese databases, including PubMed, Embase, Web of Science, Cochrane Library, China National Knowledge Infrastructure (CNKI), Wanfang Data, and China Science and Technology Journal Database (VIP). The search was performed from database inception to May 1, 2026. The search strategy was developed using combinations of terms related to three key concepts: type 2 diabetes (“Diabetes Mellitus,” “Type 2 diabetes”), TyG-BMI (“Triglyceride Glucose-Body Mass Index,” “TYG-BMI”), and cohort studies (“cohort study” or “cohort studies”). The detailed search strategies for each database are provided in Supplementary Material 2.

### 2.2 Study selection criteria

The eligibility criteria were established according to the PECOS framework, including Population, Exposure, Comparators, Outcomes, and Study design.

#### 2.2.1 Participants

The study population consisted of adults aged ≥ 18 years without a history of T2D at baseline. Studies were required to provide sufficient information regarding participant recruitment and baseline characteristics.

#### 2.2.2 Exposure

The primary exposure was TyG-BMI. The TyG-BMI index was calculated as follows:

(1) TyG = ln [triglycerides (mg/dL) × glucose (mg/dL)/2];

(2) BMI = body mass (kg)/height^2^ (m^2^);

(3) TyG-BMI = TyG × BMI.

Studies were eligible if they reported TyG-BMI as a continuous or categorical exposure. For continuous analyses, studies were required to report HRs per 1-standard deviation (SD) increase in TyG-BMI or provide sufficient information for conversion to a standardized scale. Studies reporting other increment scales were excluded unless conversion to per-SD estimates was possible. For categorical analyses, studies were required to compare the highest versus lowest TyG-BMI categories with clearly defined cutoff values.

#### 2.2.3 Comparators

For categorical analyses, the lowest TyG-BMI category was used as the reference group. For continuous analyses, effect estimates were required to represent a 1-SD increase in TyG-BMI.

#### 2.2.4 Outcomes

The primary outcome was incident T2D diagnosed according to clearly defined criteria. Eligible studies were required to report multivariable-adjusted HRs and 95% CIs for the association between TyG-BMI and incident T2D. Studies with substantial loss to follow-up without appropriate handling of missing data were excluded.

#### 2.2.5 Study design

Eligible studies included prospective or retrospective cohort studies with baseline TyG-BMI assessment and subsequent follow-up for incident T2D. Studies had to be published in peer-reviewed Chinese or English journals and provide sufficient data for effect estimation.

### 2.3 Exclusion criteria

#### 2.3.1 Types of participants

Minors or unclear age range; unclear baseline diabetes status; studies involving populations with specific diseases or clinical conditions substantially different from the general population (e.g., type 1 diabetes, secondary diabetes, gestational diabetes); insufficient information regarding population characteristics.

#### 2.3.2 Exposure

Reporting only TyG or BMI alone without the combined TyG-BMI index; inconsistent TyG calculation methods or non-standardized units that prevented valid conversion; unclear BMI definitions or unavailable categorical thresholds; TyG-BMI exposure without extractable effect estimates; unavailable HRs or insufficient data for calculating effect estimates.

#### 2.3.3 Comparators

Inadequately defined reference groups; categorical analyses without clearly defined lowest TyG-BMI category as the reference; continuous analyses without clearly defined increment scales; studies reporting only unadjusted effect estimates.

#### 2.3.4 Outcome measures

Outcomes not restricted to incident T2D; unclear or non-standardized diagnostic criteria for T2D; unavailable HRs and 95% CIs; follow-up duration < 1 year or complete loss to follow-up without description of missing data handling.

#### 2.3.5 Type of study type

Non-cohort study designs; non-peer-reviewed articles; duplicate publications; inaccessible full texts or missing key data; articles not published in Chinese or English.

### 2.4 Literature screening and data extraction

Two researchers (H. Yu and J.Y. Zhang) independently screened the retrieved records using EndNote X9 software and evaluated study eligibility according to predefined inclusion and exclusion criteria. Any disagreements were resolved through discussion and, when necessary, adjudicated by a third researcher (J. Sun) until consensus was reached. Two other researchers (B. Dai and Y.X. Chen) independently extracted data from the included studies, including author name, publication year, study country, study design, sample size, proportion of male participants, participant characteristics, follow-up duration, TyG-BMI calculation method, continuous or categorical exposure definitions, increment scale or cutoff categories, outcome assessment methods, effect estimates (HRs with 95% CIs), and adjusted covariates. The extracted data were cross-checked between researchers to ensure accuracy and consistency.

### 2.5 Quality assessment

All included studies were cohort studies, and methodological quality was assessed using the Newcastle–Ottawa Scale (NOS) (13). The NOS evaluates three domains: selection of study cohorts, comparability of cohorts, and ascertainment of outcomes, with a total score ranging from 0 to 9 points. Studies with scores ≥ 7 were considered to be of high methodological quality. The quality assessment was independently performed by two researchers (Z.R. Liu and Y. Zhang). Any disagreements were resolved through discussion and consensus.

### 2.6 Statistical analysis

Statistical analyses were performed using Review Manager 5.4.1 and Stata 16.0. Because incident T2D was a time-to-event outcome, hazard ratios (HRs) with 95% confidence intervals (CIs) were used as the effect measures to quantify the association between TyG-BMI and incident T2D. Separate meta-analyses were conducted for continuous and categorical TyG-BMI. For continuous analyses, pooled HRs were calculated per 1-SD increment in TyG-BMI. For categorical analyses, pooled HRs were calculated by comparing the highest versus lowest TyG-BMI categories.

Statistical heterogeneity was assessed using Cochran’s Q test and the I^2^ statistic. A *P*-value < 0.10 for the Q test was considered indicative of significant heterogeneity. The magnitude of heterogeneity was interpreted according to Cochrane recommendations. When substantial heterogeneity was observed, subgroup analyses were conducted to explore potential sources of variability. Subgroup analyses were performed according to sex distribution ( ≥ 50% vs. < 50% male participants), follow-up duration ( ≥ 7 vs. < 7 years), and sample size ( ≥ 9000 vs. < 9000 participants). Differences between subgroups were assessed using the subgroup difference test, with *P* < 0.05 considered statistically significant. Random-effects models were applied when substantial heterogeneity was present, whereas fixed-effects models were used when heterogeneity was low. Sensitivity analyses were performed using a leave-one-out approach by sequentially removing individual studies to evaluate the robustness of pooled estimates. Meta-regression was not performed because the number of included studies was insufficient to provide reliable estimates. Publication bias was assessed by visual inspection of funnel plots and Egger’s regression test. A *P*-value < 0.05 was considered indicative of statistically significant funnel plot asymmetry. Because fewer than 10 studies were included in each analysis, publication bias assessments were interpreted cautiously.

## 3 Results

### 3.1 Literature screening results

A total of 210 records were initially identified through database searches. After removal of 43 duplicate records, 167 records remained for title and abstract screening. Subsequently, 149 records were excluded based on title and abstract evaluation, leaving 18 articles for full-text assessment. After full-text review, 12 articles were excluded for the following reasons: duplicate or overlapping data (*n* = 2), non-cohort study design (*n* = 4), effect size reported as OR only (*n* = 3), inconsistent effect measures for continuous-variable analyses (*n* = 2), and ineligible outcome (*n* = 1). Finally, six cohort studies were included in this meta-analysis (14–19). The study selection process is illustrated in Figure 1.

**FIGURE 1:**
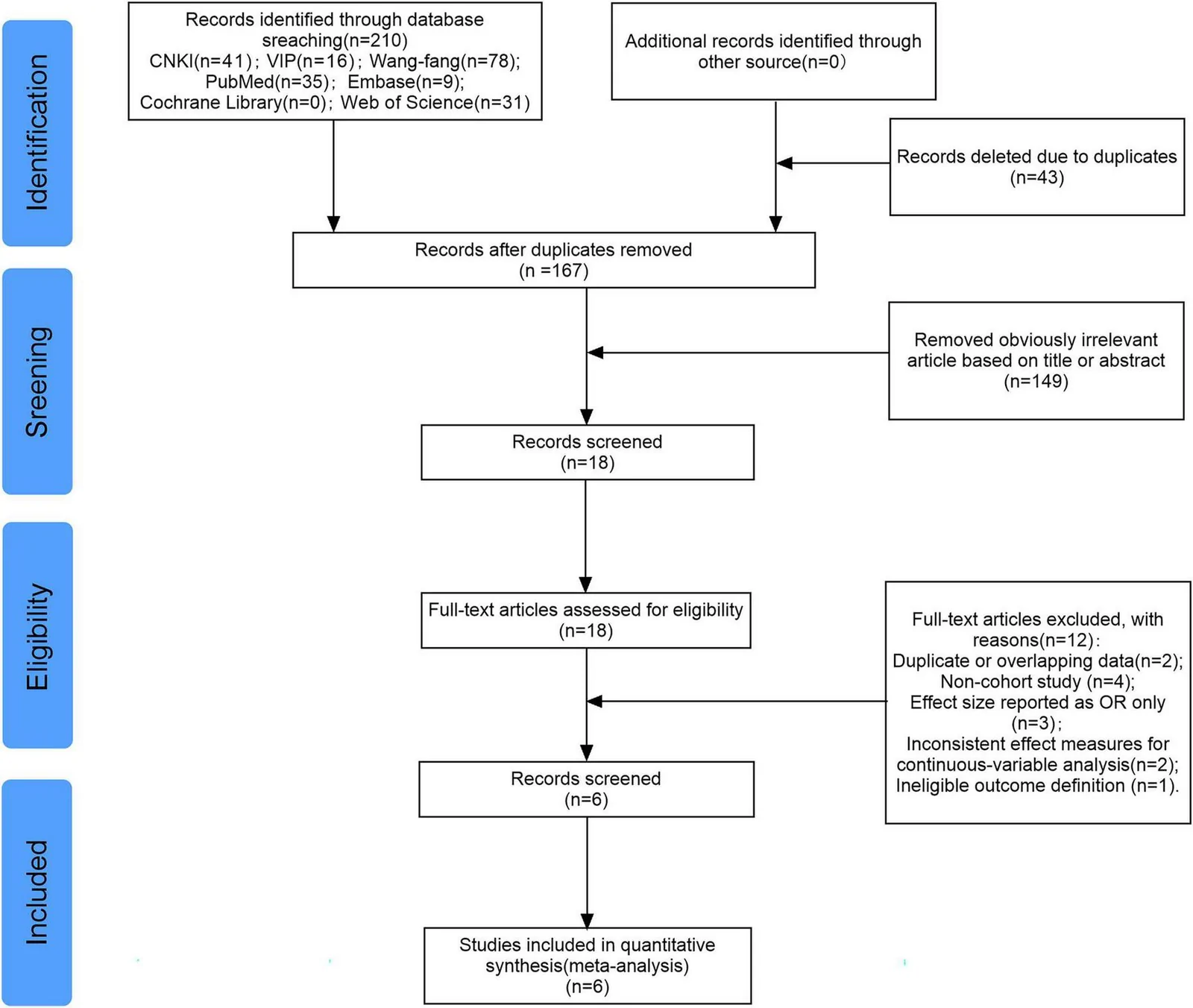
Flowchart of study selection strategy.

### 3.2 Basic information of the included studies

A total of six cohort studies published between 2021 and 2025 were included in this meta-analysis. The included studies were conducted in China (*n* = 5) and Japan (*n* = 1), with sample sizes ranging from 699 to 116,661 participants. Overall, these studies included 176,127 participants and investigated the association between TyG-BMI and incident type 2 diabetes. The follow-up duration ranged from 3.1 to 34 years. TyG-BMI was assessed using both continuous and categorical approaches, and multivariable-adjusted effect estimates were reported based on study-specific adjustment models, including demographic characteristics, lifestyle factors, metabolic parameters, and medical history. Detailed characteristics of the included studies are summarized in Table 1.

**TABLE 1:** Basic characteristics of the included studies.

Number	Study	Country	Study design	Male (%)	Age (years)	Study time (years)	Number of participants	TyG-BMI index condition	Type of outcome	Variables adjusted
The association of TyG-BMI index with risk of type 2 diabetes										
1	Zhang et al. (19)	China	Cohort study	40.22%	Median age: 57 years	7 years	29351	Continuous; categorized	Type 2 diabetes	Age, gender, educational attainment, smoking, alcohol consumption, SBP, DBP, TC, LDL-C, and family history of diabetes
2	Huang et al. (14)	China	Cohort study	46.76%	Average age: 51.9 years	7 years	7408	Continuous; categorized	Type 2 diabetes	Age, gender, residence, marital status, education level, height, weight, SBP, DBP, TC, HDL-C, LDL-C, UA, smoking, alcohol consumption, hypertension, heart disease, dyslipidemia, hyperuricemia, overweight and obesity
3	Wang et al. (17)	China	Cohort study	91.18%	Age < 60 years	5 years	6544	Continuous; categorized	Type 2 diabetes	Age, gender, educational attainment, marital status, family history of diabetes, smoking, alcohol consumption, diet, physical activity, TG, LDL-C, exposure to carbon monoxide, dust, high temperatures, noise, shift work
4	Wang et al. (18)	China	Cohort study	53.80%	Average age: 44.07 ± 12.93 years	3.1 ± 0.95 years	116,661	Continuous; categorized	Type 2 diabetes	Age, Gender, SBP, DBP, FPG, TG, HDL-C, LDL-C, ALT, AST, BUN, Scr, Smoking Status, Alcohol Consumption Status, Family History of Diabetes, and Height
5	Wang et al. (16)	China	Cohort study	52.50%	Average age: 44.67 ± 0.34 years	34 years	699	Categorized	Type 2 diabetes	Age, gender, smoking status, SBP, TC, and lifestyle intervention
6	Song et al. (15)	Japan	Cohort study	54.52%	Average age: 43.71 ± 8.90 years	13 years	15,464	Categorized	Type 2 diabetes	Age, Gender, HDL, SBP, DBP, TC, triglycerides, alcohol consumption, smoking, exercise habits, fatty liver, ALT, AST, GGT

SBP, Systolic Blood Pressure; DBP, Diastolic Blood Pressure; TC, Total Cholesterol; TG, triglycerides; HDL-C, High-Density Lipoprotein Cholesterol; LDL-C, Low-Density Lipoprotein Cholesterol; HbA1c, glycated hemoglobin A1c; FPG, Fasting Plasma Glucose; ALT, Alanine Aminotransferase; AST, Aspartate Aminotransferase; BUN, Blood Urea Nitrogen; UA, Uric Acid; Scr, Serum Creatinine.

### 3.3 Quality assessment

The methodological quality of the included studies was assessed using the Newcastle–Ottawa Scale (NOS). Six cohort studies were evaluated across three domains: selection of study cohorts, comparability of cohorts, and ascertainment of outcomes. Each item was scored as “1” when the predefined quality criterion was satisfied and “0” otherwise, with a maximum score of 9 points. The NOS scores of the included studies ranged from 7 to 9 points, indicating generally high methodological quality. Two studies received 9 points, three studies received 8 points, and one study received 7 points. Most included studies demonstrated satisfactory quality in cohort selection and comparability domains. Some limitations were related to follow-up adequacy and completeness of outcome information. Detailed results of the NOS assessment are presented in Table 2.

**TABLE 2:** Newcastle–Ottawa Scale assessment of the included cohort studies.

Study (publication year)	Selection of cohorts			Comparability of cohorts			Outcome of cohorts			Total
	Representativeness of the exposed cohort	Selection of the non-exposed cohort	Ascertainment of exposure	Outcome not present at baseline	Control for age	Control for other confounding factors	Assessment of outcome	Sufficient follow-up duration	Adequacy of follow-up of cohorts	
Zhang et al. (19)	1	1	1	1	1	1	1	1	0	8
Huang et al. (14)	1	1	1	1	1	1	1	1	1	9
Wang et al. (17)	1	1	1	1	1	1	1	1	0	8
Song et al. (15)	1	1	1	1	1	1	1	1	0	8
Wang et al. (18)	1	1	1	1	1	1	1	0	0	7
Wang et al. (16)	1	1	1	1	1	1	1	1	1	9

### 3.4 Meta-analysis results

This study employed a random-effects model to conduct meta-analyses, including analyses of data based on the continuous distribution of the TyG-BMI index across 4 studies and analyses of pre-defined categorical TyG-BMI data across 6 studies.

#### 3.4.1 Meta-analysis of continuous variables

Four cohort studies reporting the association between continuous TyG-BMI and incident type 2 diabetes were included in the meta-analysis. All included studies reported HRs per 1-standard deviation (SD) increment in TyG-BMI, and the increment scale was consistent across studies (Supplementary Material 3). Due to significant heterogeneity among studies (I^2^ = 87%, *P* < 0.001), a random-effects model was applied. The pooled analysis demonstrated that each 1-SD increment in TyG-BMI was significantly associated with a higher risk of incident type 2 diabetes (HR = 1.68, 95% CI: 1.50–1.88, *P* < 0.001; Figure 2).

**FIGURE 2:**
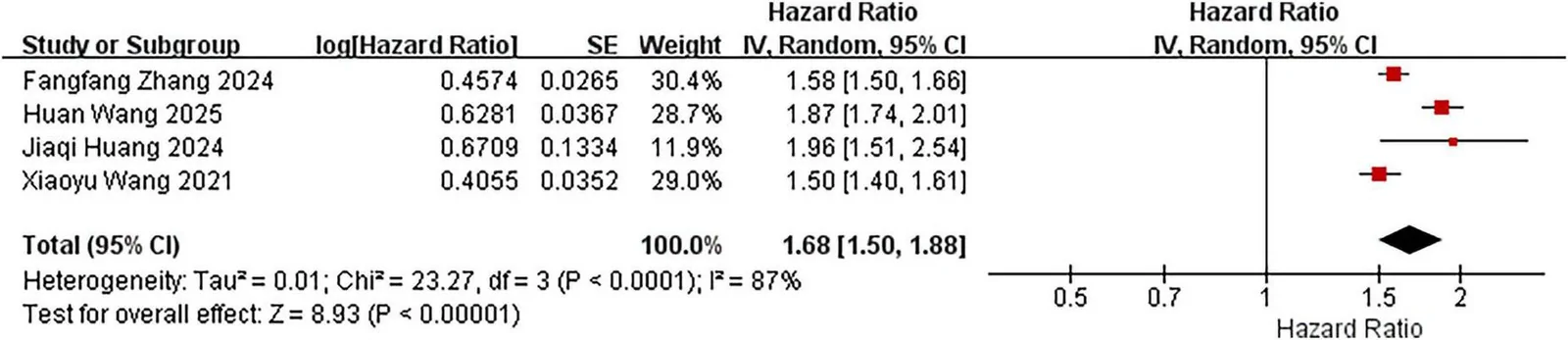
Forest plot of continuous variables.

Stratified subgroup analyses were performed to explore potential sources of heterogeneity. According to the proportion of male participants, studies were divided into ≥ 50% and < 50% male subgroups. The pooled HR was 1.67 (95% CI: 1.33–2.09, *P* < 0.001) in the ≥ 50% male subgroup, with considerable heterogeneity (I^2^ = 96%), and 1.69 (95% CI: 1.39–2.05, *P* < 0.001) in the < 50% male subgroup, with substantial heterogeneity (I^2^ = 59%). No significant difference was observed between these two subgroups (χ^2^ = 0.01, df = 1, *P* = 0.94; Figure 3A). When stratified by follow-up duration, studies with follow-up periods ≥ 7 years showed a pooled HR of 1.69 (95% CI: 1.39–2.05, *P* < 0.001), with substantial heterogeneity (I^2^ = 59%), whereas studies with follow-up periods < 7 years showed a pooled HR of 1.67 (95% CI: 1.33–2.09, *P* < 0.001), with considerable heterogeneity (I^2^ = 96%). No significant subgroup difference was detected (χ^2^ = 0.01, df = 1, *P* = 0.94; Figure 3B). According to sample size, studies were categorized into ≥ 9000 and < 9000 participants. The pooled HR was 1.54 (95% CI: 1.45–1.63, *P* < 0.001) among studies with ≥ 9000 participants, with substantial heterogeneity (I^2^ = 50%). In contrast, studies with < 9000 participants showed a pooled HR of 1.88 (95% CI: 1.75–2.01, *P* < 0.001), with no observed heterogeneity (I^2^ = 0%). A statistically significant difference was observed between the two sample-size subgroups (χ^2^ = 19.09, df = 1, *P* < 0.001; Figure 3C).

**FIGURE 3:**
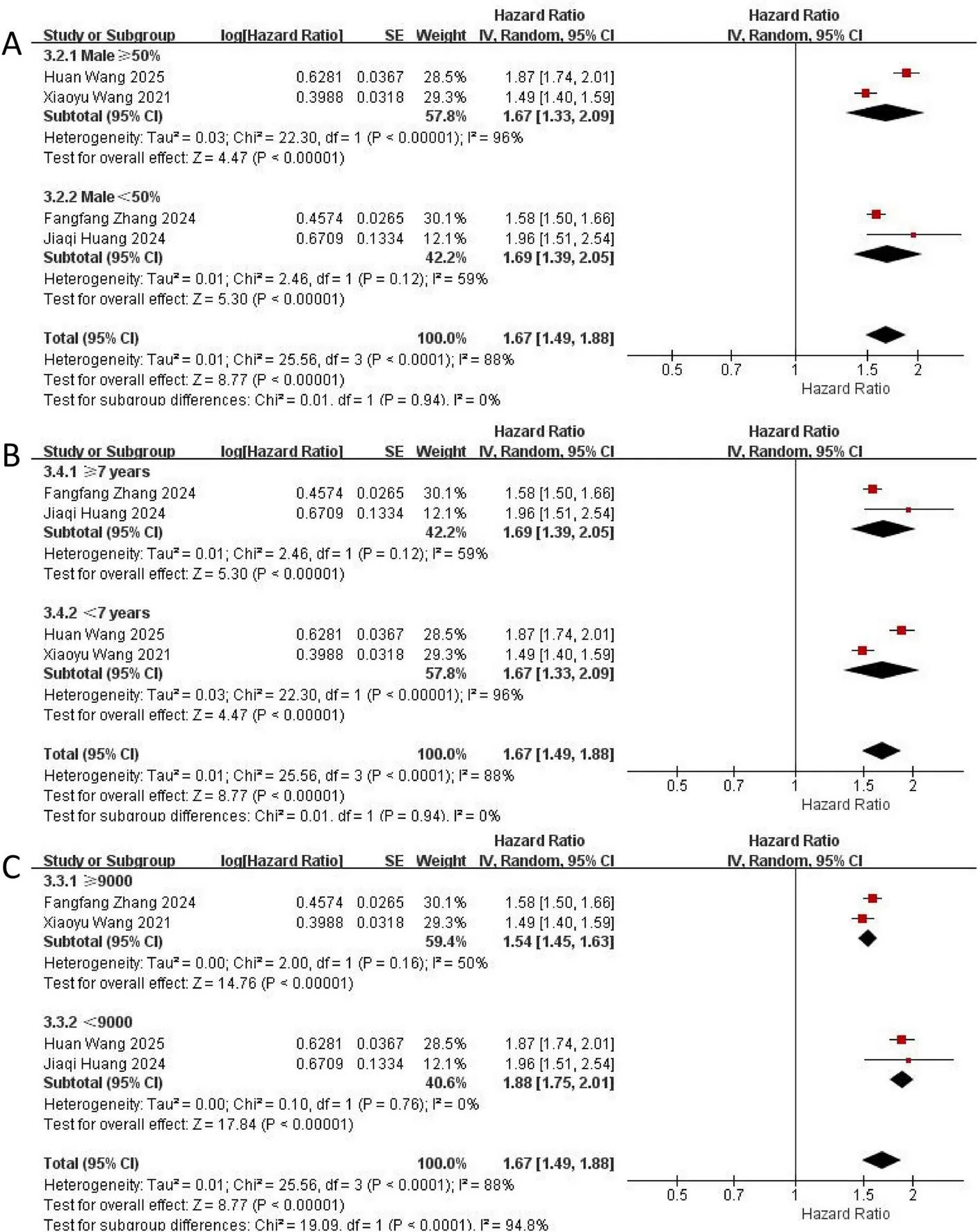
Forest plot of continuous variable subgroup analysis for panel **(A)** subgroup analysis according to proportion of male participants, **(B)** subgroup analysis according to follow-up time, **(C)** subgroup analysis according to the sample size.

Overall, subgroup analyses indicated that sex distribution and follow-up duration did not significantly explain the observed heterogeneity. However, sample size may partly explain the variability in effect estimates among studies.

#### 3.4.2 Meta-analysis of categorical variables

Six cohort studies reporting the association between categorical TyG-BMI and incident type 2 diabetes were included in the meta-analysis. Significant heterogeneity was observed among the included studies (I^2^ = 89%, *P* < 0.001); therefore, a random-effects model was applied. The pooled analysis showed that individuals in the highest TyG-BMI categories had a significantly increased risk of incident type 2 diabetes compared with those in the lowest categories (HR = 3.35, 95% CI: 2.24–5.03, *P* < 0.001; Figure 4).

**FIGURE 4:**
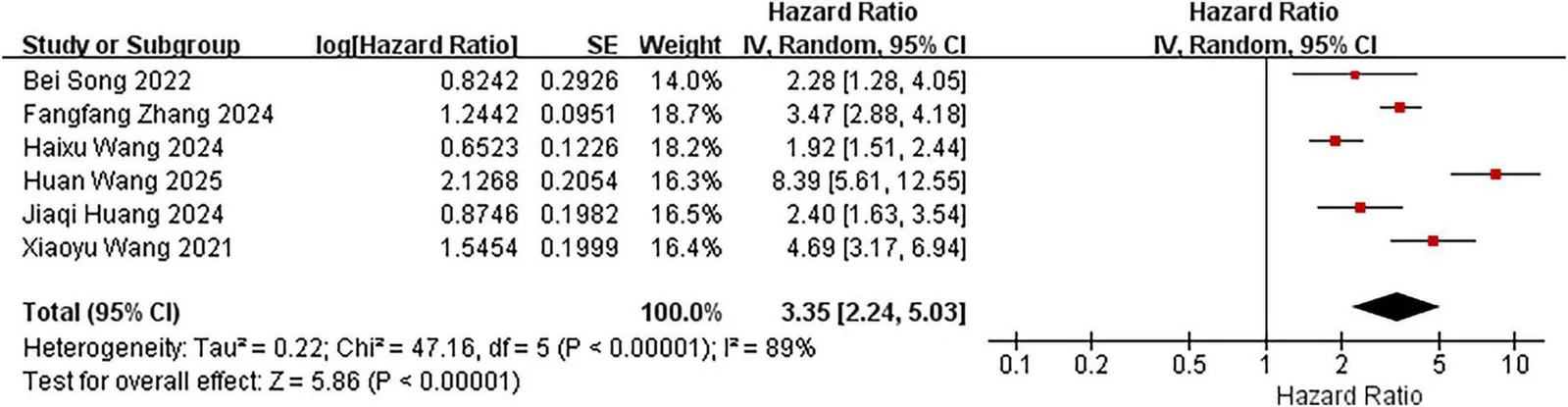
Forest plot of categorical variables.

Stratified subgroup analyses were performed to explore potential sources of heterogeneity. According to the proportion of male participants, the pooled HR was 3.62 (95% CI: 1.76–7.44, *P* = 0.0005) in studies with ≥ 50% male participants, with considerable heterogeneity (I^2^ = 93%), whereas the pooled HR was 3.01 (95% CI: 2.11–4.28, *P* < 0.001) in studies with < 50% male participants, with substantial heterogeneity (I^2^ = 65%). No significant difference was observed between these two subgroups (χ^2^ = 0.20, df = 1, *P* = 0.65; Figure 5A). When stratified by follow-up duration, studies with follow-up periods ≥ 7 years showed a pooled HR of 2.50 (95% CI: 1.77–3.53, *P* < 0.001), with substantial heterogeneity (I^2^ = 81%). In contrast, studies with follow-up periods < 7 years showed a pooled HR of 6.22 (95% CI: 3.49–11.10, *P* < 0.001), with considerable heterogeneity (I^2^ = 77%). A significant difference was observed between these two subgroups (χ^2^ = 7.08, df = 1, *P* = 0.008), suggesting that follow-up duration may partially contribute to the observed heterogeneity (Figure 5B). According to sample size, studies were categorized into ≥ 9000 and < 9000 participants. The pooled HR was 3.51 (95% CI: 2.60–4.73, *P* < 0.001) among studies with ≥ 9000 participants, with substantial heterogeneity (I^2^ = 53%). For studies with < 9000 participants, the pooled HR was 3.35 (95% CI: 1.42–7.91, *P* = 0.006), with considerable heterogeneity (I^2^ = 95%). No significant difference was observed between the two sample-size subgroups (χ^2^ = 0.01, df = 1, *P* = 0.92; Figure 5C).

**FIGURE 5:**
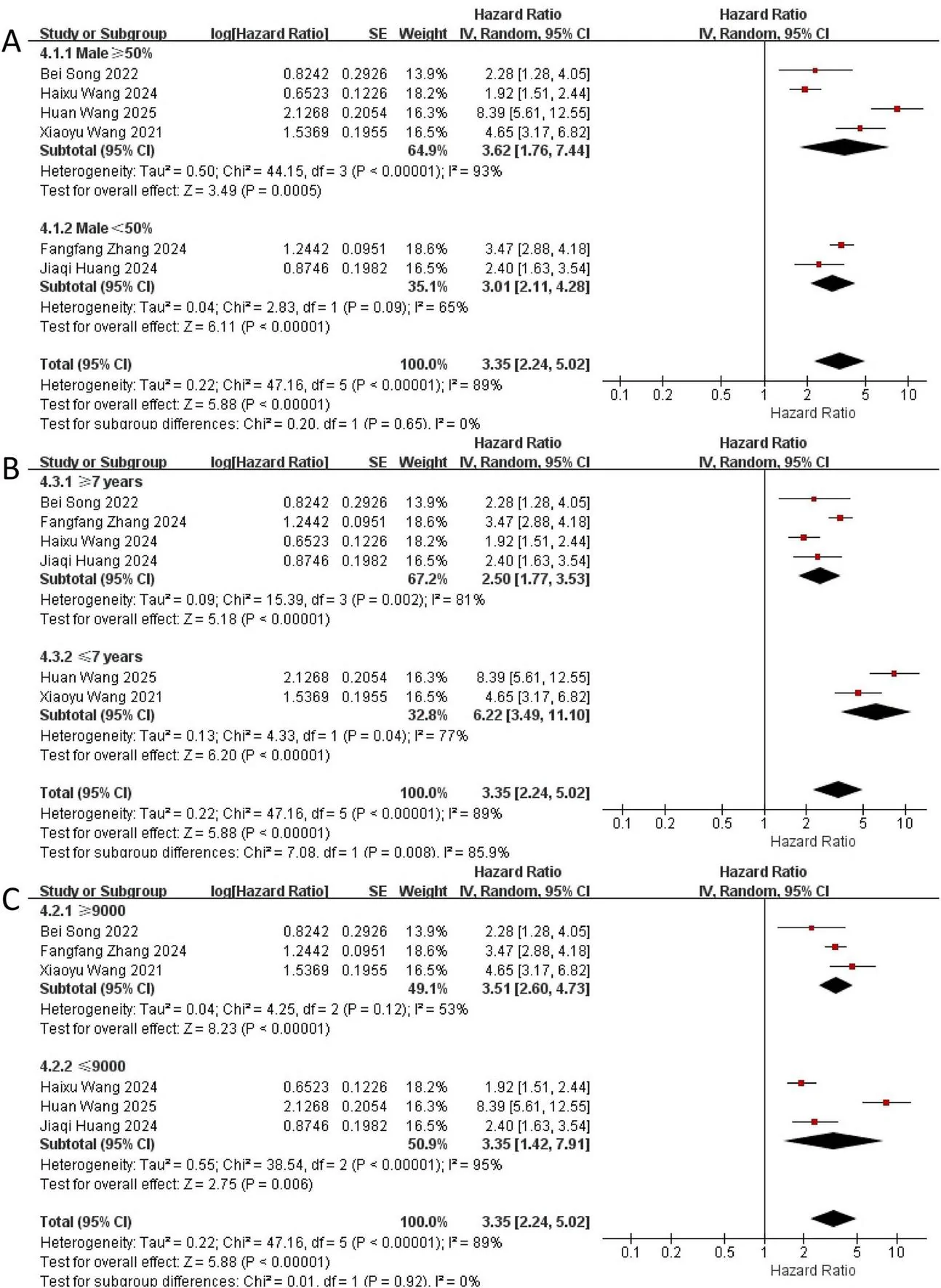
Forest plot of categorical variable subgroup analysis for panel **(A)** subgroup analysis according to proportion of male participants, **(B)** subgroup analysis according to follow-up time, **(C)** subgroup analysis according to the sample size.

Overall, subgroup analyses suggested that follow-up duration may partially explain the observed heterogeneity, with stronger associations observed in studies with shorter follow-up periods ( < 7 years). In contrast, sex distribution and sample size did not significantly explain the heterogeneity among studies. Residual heterogeneity remained in several subgroup analyses, indicating that other study-level characteristics may also contribute to between-study variability.

### 3.5 Publication bias analysis

Publication bias was assessed using funnel plots with pseudo 95% confidence limits and Egger’s regression test. For the continuous-variable analysis including four cohort studies (Figure 6A), the funnel plot showed an approximately symmetrical distribution, and Egger’s test did not indicate statistically significant asymmetry (intercept *P* = 0.559). Similarly, for the categorical-variable analysis including six cohort studies (Figure 6B), the funnel plot appeared relatively symmetrical, with no statistically significant evidence of asymmetry according to Egger’s test (intercept *P* = 0.710). However, because fewer than 10 studies were included in each analysis, the power of funnel plot interpretation and Egger’s regression test was limited. Therefore, the absence of statistically significant asymmetry should not be interpreted as definitive evidence that publication bias is absent, and the possibility of publication bias cannot be completely excluded.

**FIGURE 6:**
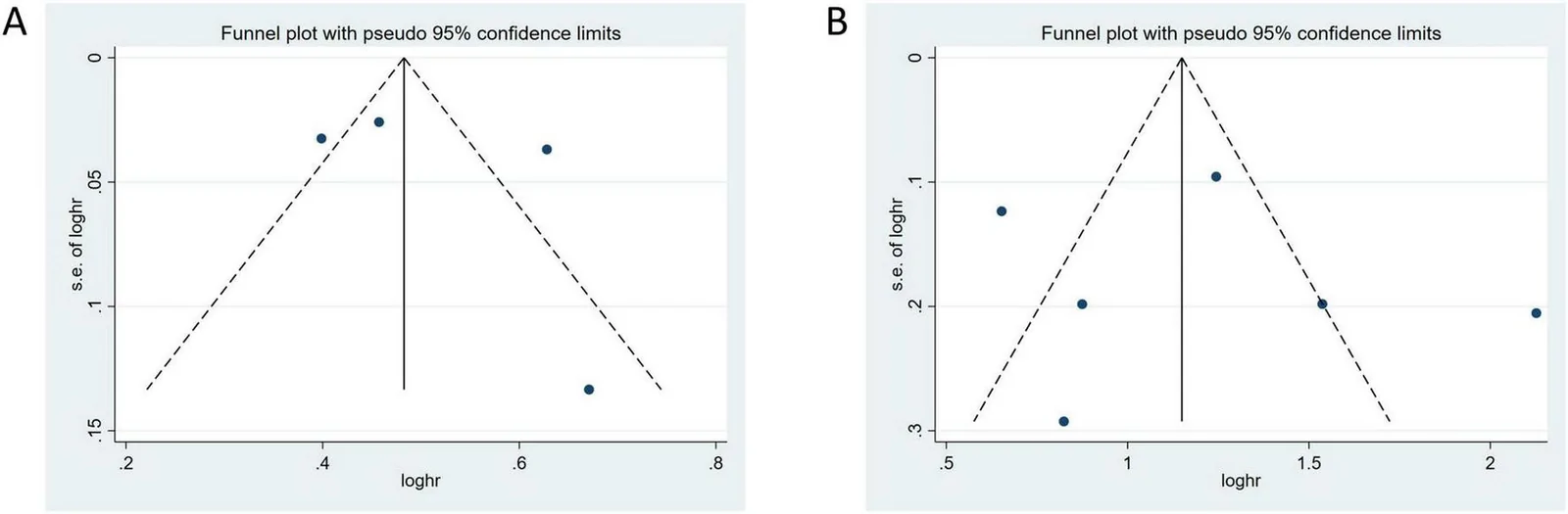
Funnel plots for panel **(A)** continuous variables, **(B)** categorical variables.

### 3.6 Sensitivity analysis

The leave-one-out sensitivity analysis demonstrated that the pooled effect estimates remained statistically significant after sequential exclusion of individual studies, indicating that the overall findings were robust. In the continuous-variable analysis, exclusion of the study by Huan Wang et al. reduced heterogeneity from I^2^ = 87% to I^2^ = 54%, while the pooled HR remained significant (HR = 1.57, 95% CI: 1.46–1.69), suggesting that this study may have contributed to the observed heterogeneity. This finding may be related to differences in study population characteristics, as Huan Wang et al. focused on steelworkers with specific occupational exposures, whereas the other included studies mainly involved general community populations. Occupational factors, such as shift work and environmental exposures, may influence metabolic profiles and diabetes susceptibility, potentially affecting the association between TyG-BMI and incident T2D. In the categorical-variable analysis, exclusion of any individual study did not substantially change the pooled estimates, with HRs ranging from 2.81 to 3.80, and all results remained statistically significant (all *P* < 0.001). Although removal of Huan Wang et al. slightly reduced heterogeneity (from I^2^ = 89% to I^2^ = 82%), substantial heterogeneity remained, suggesting that multiple study-level factors may contribute to variability among the included studies. Detailed results of the sensitivity analyses are presented in Tables 3, 4.

**TABLE 3:** Sensitivity analysis of continuous variables.

Dataset excluded	HR	95%CI	I^2^%	*P* for effect
Zhang et al. (19)	1.74	[1.44, 2.09]	90	*P* < 0.001
Wang et al. (17)	1.57	[1.46, 1.69]	54	*P* < 0.001
Huang et al. (14)	1.64	[1.46, 1.85]	91	*P* < 0.001
Wang et al. (18)	1.76	[1.52, 2.04]	87	*P* < 0.001

**TABLE 4:** Sensitivity analysis of categorical variables.

Dataset excluded	HR	95%CI	I^2^%	*P* for effect
Song et al. (15)	3.58	[2.28, 5.61]	91	*P* < 0.001
Zhang et al. (19)	3.33	[1.89, 5.88]	91	*P* < 0.001
Wang et al. (16)	3.80	[2.54, 5.69]	84	*P* < 0.001
Wang et al. (17)	2.81	[2.01, 3.92]	82	*P* < 0.001
Huang et al. (14)	3.59	[2.24, 5.75]	91	*P* < 0.001
Wang et al. (18)	3.14	[1.98, 4.97]	91	*P* < 0.001

## 4 Discussion

Research indicates that the global prevalence of T2D among adults has surpassed 10%, exhibiting an annual upward trend toward younger age groups. It has emerged as one of the primary chronic noncommunicable diseases threatening human health (20). As a metabolic disorder triggered by multiple factors including genetics and environment, the core pathophysiological features of T2D are IR and pancreatic β-cell dysfunction (21). IR not only initiates the onset and progression of T2D but is also closely associated with metabolic dysregulation such as obesity and dyslipidemia, significantly increasing the risk of diabetic complications (22). Previous studies have demonstrated that the TyG index is a useful surrogate marker for assessing IR, while BMI remains the classic parameter reflecting systemic obesity (8, 10, 23). This meta-analysis represents the first systematic integration of available cohort evidence and supports a significant positive association between elevated TyG-BMI and increased T2D risk. It provides novel epidemiological evidence supporting the association between TyG-BMI and incident T2D risk.

Traditional risk factors for T2D include age, family history, central obesity, hypertension, dyslipidemia, physical inactivity, and unhealthy diet (24, 25). Currently, T2D is primarily diagnosed clinically through fasting blood glucose, 2-h postprandial glucose, and glycated hemoglobin levels. However, these indicators often only show abnormalities after glucose metabolism disorders have progressed to a certain stage, making early disease warning difficult to achieve (26). Furthermore, while the oral glucose tolerance test (OGTT) serves as the gold standard for diagnosing glucose metabolism abnormalities, it suffers from limitations such as cumbersome procedures, time-consuming administration, and poor patient compliance, rendering it unsuitable for large-scale population screening (27). In contrast, the TyG-BMI index calculation requires only fasting blood glucose, fasting triglycerides, and height-weight data. It offers advantages of simplicity, non-invasiveness, and low cost, suggesting potential utility in population-based metabolic risk assessment. More importantly, the TyG-BMI index simultaneously reflects both IR and obesity status—the two key drivers of T2D pathogenesis. Therefore, TyG-BMI may provide additional information for identifying individuals at higher metabolic risk, although its incremental predictive value beyond established risk factors requires further validation. Identification of individuals with elevated TyG-BMI may help characterize populations with higher metabolic risk and warrants further investigation.

Although the association between the TyG-BMI index and the risk of developing T2D has been consistently observed in this meta-analysis, its specific mechanism of action remains incompletely elucidated. It is speculated to be closely related to multiple pathways, including IR, dyslipidemia, chronic inflammation, and oxidative stress. First, the TyG index itself serves as an effective indicator of IR in the liver and peripheral tissues. Elevated BMI, representing obesity–particularly visceral fat accumulation–urther exacerbates IR (7, 28). Visceral adipose tissue secretes numerous adipokines, such as leptin, adiponectin, and resistin, which interfere with insulin signaling pathways, inhibit the phosphorylation of insulin receptor substrates, and reduce insulin sensitivity (29). Concurrently, excessive proliferation of adipose tissue in obesity induces local hypoxia, leading to macrophage infiltration and activation of inflammatory pathways like nuclear factor κB (NF-κB). This promotes the release of pro-inflammatory cytokines such as tumor necrosis factor-α (TNF-α) and interleukin-6 (IL-6) (30–32). These inflammatory mediators not only directly impair pancreatic β-cell function but also exacerbate IR, creating a vicious cycle that may contribute to the development of T2D.

In the present meta-analysis, six cohort studies involving 176,127 participants were included to investigate the association between TyG-BMI and incident T2D using both continuous and categorical approaches. In the continuous-variable analysis, four studies reporting HRs per 1-standard deviation (SD) increase in TyG-BMI were included. The pooled results demonstrated that each 1-SD increase in TyG-BMI was significantly associated with a higher risk of incident T2D (HR = 1.68, 95% CI: 1.50–1.88, *P* < 0.001), although substantial heterogeneity was observed (I^2^ = 87%). In the categorical-variable analysis, individuals with higher TyG-BMI categories had a significantly increased risk of incident T2D compared with those with lower categories (HR = 3.35, 95% CI: 2.24–5.03, *P* < 0.001), with considerable heterogeneity among studies (I^2^ = 89%). These findings consistently indicate an association between elevated TyG-BMI and increased risk of developing T2D.

Subgroup analyses were conducted to explore potential sources of heterogeneity. In the continuous-variable analysis, stratification according to sex distribution, follow-up duration, and sample size did not demonstrate significant differences between subgroups, although sample size appeared to partially contribute to variability in effect estimates. In the categorical-variable analysis, follow-up duration appeared to influence the magnitude of the association, with stronger effect estimates observed among studies with shorter follow-up periods ( < 7 years; HR = 6.22, 95% CI: 3.49–11.10) compared with those with longer follow-up periods ( ≥ 7 years; HR = 2.50, 95% CI: 1.77–3.53). This difference may be related to variations in baseline metabolic characteristics, population composition, or follow-up-related factors, although the underlying mechanisms remain unclear.

Sensitivity analyses demonstrated that the pooled estimates were stable, as sequential exclusion of individual studies did not substantially change the overall effect sizes. Funnel plots and Egger’s tests showed no statistically significant evidence of publication bias; however, these findings should be interpreted cautiously because the number of included studies was limited.

Despite the consistent association between elevated TyG-BMI and increased risk of incident T2D, substantial heterogeneity remained among the included studies. Several factors may contribute to this variability. First, differences in study populations may partly explain the observed heterogeneity. Although all included studies were conducted in Asian populations, variations existed in baseline characteristics, including age distribution, sex composition, occupational background, and metabolic status. For example, one study focused on steelworkers with specific occupational exposures, whereas the remaining studies mainly included general community-based populations, which may have influenced metabolic profiles and diabetes susceptibility.

Second, differences in TyG-BMI assessment and analytical approaches may have contributed to variability in effect estimates. Although all included studies applied the conventional TyG-BMI calculation framework and continuous analyses were harmonized using per 1-SD increments, differences in population distributions and categorical classification criteria (such as tertiles or quartiles) may have affected the comparability of results.

Third, follow-up duration may influence the magnitude of the observed association. Stronger associations were observed in studies with shorter follow-up periods, which may partly reflect reverse causality. Individuals with undiagnosed diabetes or early-stage metabolic abnormalities at baseline may already have elevated TyG-BMI levels, leading to an overestimation of the association during early follow-up. Although all included studies excluded participants with diagnosed diabetes at baseline, subclinical metabolic disturbances could not be completely excluded.

Finally, variations in covariate adjustment strategies may have contributed to residual heterogeneity. Although all studies reported multivariable-adjusted HRs, the selected confounders differed across studies, including demographic characteristics, lifestyle factors, metabolic parameters, and occupational exposures. These differences may influence the extent of residual confounding and affect the comparability of pooled estimates. Given the limited number of included studies, meta-regression was not performed because its reliability would be limited. Therefore, residual heterogeneity should be interpreted cautiously, and future large-scale studies using standardized TyG-BMI assessment protocols and more consistent analytical approaches are warranted.

Overall, this meta-analysis supports a positive association between higher TyG-BMI levels and incident T2D risk. However, given the observational nature of current evidence and the remaining heterogeneity among studies, these findings should be interpreted cautiously. Further large-scale prospective studies involving diverse populations are warranted to clarify the potential role of TyG-BMI in diabetes risk assessment.

## 5 Strengths and limitations

This meta-analysis has several strengths. First, to our knowledge, this is the first systematic review and meta-analysis to comprehensively evaluate the association between TyG-BMI and incident type 2 diabetes, providing quantitative evidence regarding the relationship between this metabolic index and future diabetes occurrence. Second, only cohort studies with relatively high methodological quality were included, which enhanced the credibility of the findings. Third, both continuous and categorical analyses of TyG-BMI were performed, and subgroup analyses and sensitivity analyses were conducted to explore the robustness and potential sources of heterogeneity.

Several limitations should also be considered. First, although all included studies were cohort studies, the observational nature of the evidence prevents causal inference. The possibility of reverse causality cannot be completely excluded, as individuals with undiagnosed diabetes or early metabolic abnormalities at baseline may already have elevated TyG-BMI levels, particularly in studies with shorter follow-up periods. Second, substantial heterogeneity existed among studies. Although the TyG-BMI calculation framework was generally consistent, differences in population characteristics, laboratory measurements, categorical cutoff definitions, follow-up duration, and covariate adjustment strategies may have contributed to variability among effect estimates. Different adjustment models across studies may also have resulted in residual confounding and reduced comparability. Third, all included studies were conducted in Asian populations (China and Japan), limiting the generalizability of the findings to other populations. Finally, the limited number of eligible studies reduced the statistical power of subgroup analyses and publication bias assessments, and potential publication bias cannot be completely excluded.

## 6 Conclusion

In summary, this meta-analysis demonstrates that higher TyG-BMI levels are significantly associated with an increased risk of incident T2D. As a composite indicator reflecting insulin resistance and obesity-related metabolic abnormalities, TyG-BMI may have potential value for identifying individuals at increased metabolic risk. However, causal relationships cannot be established based on current observational evidence, and the clinical utility of TyG-BMI for T2D prediction and prevention requires further validation in diverse populations and well-designed prospective studies.

## Data Availability

The original contributions presented in this study are included in this article/Supplementary material, further inquiries can be directed to the corresponding author.
